# Case Report: A new noninvasive device-based treatment of a mesencephalic H3 K27M glioma

**DOI:** 10.3389/fonc.2025.1626516

**Published:** 2025-11-05

**Authors:** Santosh A. Helekar, Omkar B. Ijare, Martyn A. Sharpe, Kumar Pichumani, David S. Baskin

**Affiliations:** ^1^ Department of Neurosurgery, Houston Methodist Hospital and Houston Methodist Research Institute, Houston, TX, United States; ^2^ Department of Neurological Surgery, Weill Cornell Medical College, New York, NY, United States; ^3^ Translational Biomagnetics and Neurometry Program, Houston Methodist Research Institute, Houston, TX, United States; ^4^ Kenneth R. Peak Center for Brain and Pituitary Tumor Treatment and Research, Houston Methodist Hospital, Houston, TX, United States; ^5^ Department of Neurosurgery, Texas A & M Medical School, College Station, TX, United States

**Keywords:** spinning oscillating magnetic fields, magnetic resonance imaging, contrast-enhanced tumor, compassionate use treatment, magnetic resonance spectroscopy, wearable anti-cancer device

## Abstract

Brainstem gliomas have a poor prognosis and ineffective therapeutic options. We have developed a noninvasive device called an Oncomagnetic device that produces selective oncolysis of gliomas *in vitro* and marked reduction of contrast-enhanced tumor (CET) volume in end-stage recurrent glioblastoma (GBM) patients. Here we report Oncomagnetic treatment (OMT) of a 28-year-old woman who had undergone partial surgical excision and radiotherapy of a H3 K27M midline glioma in the mesencephalon and pons. OMT initiated after the first recurrence of the tumor was well tolerated for more than 694 days by the patient. There was near-complete regression of the CET at 145 days with symptomatic relief and a partial regression at 554 days after an apparent progression at 518 days. OMT was discontinued after 694 days because of hospital admission due to injuries from a fall and disease progression, which then led to her death. These findings demonstrate the potential of a new effective, nontoxic, and noninvasive wearable device-based treatment for the deadly diffuse midline glioma.

## Introduction

Diffuse midline gliomas (DMG), previously known as diffuse intrinsic pontine gliomas (DIPG) are midline malignant brain tumors with poor prognosis and inadequate treatment options (1). They occur most commonly in children between the ages of 6 and 8 years, constituting nearly 50% of all high-grade childhood gliomas (2). In ~80% of high-grade tumors in children molecular analysis has demonstrated a lysine to methionine substitution at codon 27 (K27M) in histone H3 variants, H3F3A (~75%) and HIST1H3B (~25%) (3). This mutation was previously thought to be confined to pediatric gliomas. However, it is now found to also occur in adults (4). H3 K27M mutation is present in 80% of DMGs, and other high-grade gliomas, including in other regions, such as the thalamus (5).

In terms of the typical progression of H3 K27M diffuse midline glioma, the tumor originates in central nervous system midline structures such as pons, midbrain, thalamus, spinal cord, cerebellum or medulla oblongata and infiltrates into other areas, also extending along white matter tracts (6). While children between the ages of 5–15 years are likely to be affected, occurrence in adults in the 20 – 60-year age range is also seen (7). Symptoms, such as pain, weakness, ataxia, spasticity and loss of sensations depend on the location of the tumor and worsen over several weeks or months. Edema and increased intracranial pressure with further progression produce headaches, nausea and vomiting. In terms of the course after treatment, some improvement due to radiation therapy occurs over a period of 1–3 months. This is followed by disease progression over 3–9 months leading to a terminal phase and death typically within a year from diagnosis in children, but within a longer period in adults.

As far as treatment of DMG is concerned, Temozolomide in combination with radiation therapy shows no improvement in overall survival (8). Radiation therapy is standard of care, but it produces only a transient benefit in terms of neurologic deficits involving reduced use of steroids in symptomatic patients (9) and, at best, a modest prolongation of survival (10). While there are several new drugs being tested to develop a targeted treatment regimen, it is believed that an optimal treatment strategy might require combination therapy (10). Recently, the U.S. Food and Drug Administration (FDA) has approved a D2 dopamine receptor antagonist dordaviprone (Modeyso) as treatment for DMG (https://www.fda.gov/drugs/resources-information-approved-drugs/fda-grants-accelerated-approval-dordaviprone-diffuse-midline-glioma). As far as immunotherapy is concerned, DMGs are characterized by a limited amount of immune cell infiltration (10), and tumor cells and infiltrating macrophages release fewer cytokines than glioblastoma (GBM) (11).

Because our preclinical and clinical studies with a new noninvasive wearable device known as the Oncomagnetic device (OMD) have shown promising results in GBM (12–14), we tested the safety and efficacy of this device in a single DMG patient enrolled in an Expanded Access Program (EAP) treatment protocol. This device generates spinning oscillating magnetic fields (sOMF) by rotating strong permanent magnets that are attached to a helmet worn by the patient (12). Our preclinical studies with sOMF stimulation have demonstrated strong selective anticancer effects in patient derived GBM cells (13) and a syngeneic mouse model, with no toxicity in cultured normal cells and healthy wildtype mice (15). The mechanism of action of sOMF is completely different from FDA-approved tumor treating field device (Optune^®^) therapy used in treating GBM, which unlike sOMF, is on tubulin dimers and cell division (16). sOMF disrupts electron transport in the mitochondrial respiratory chain, e.g., transiently in Complex II succinate dehydrogenase and persistently in Complex I ubiquinone oxidoreductase, causing elevation of reactive oxygen species (ROS) and triggering oncolysis (13, 14).

In this case report we present evidence that FDA-approved compassionate use Oncomagnetic treatment (OMT) of the first adult patient with DMG was well tolerated for more than 694 days and produced a near-complete regression of an untreatable brainstem malignant glioma with an H3 K27M mutation after its first recurrence as a contrast-enhanced tumor (CET) and a partial regression after its apparent second regression.

## Methods

### Patient

**Figure 1:**
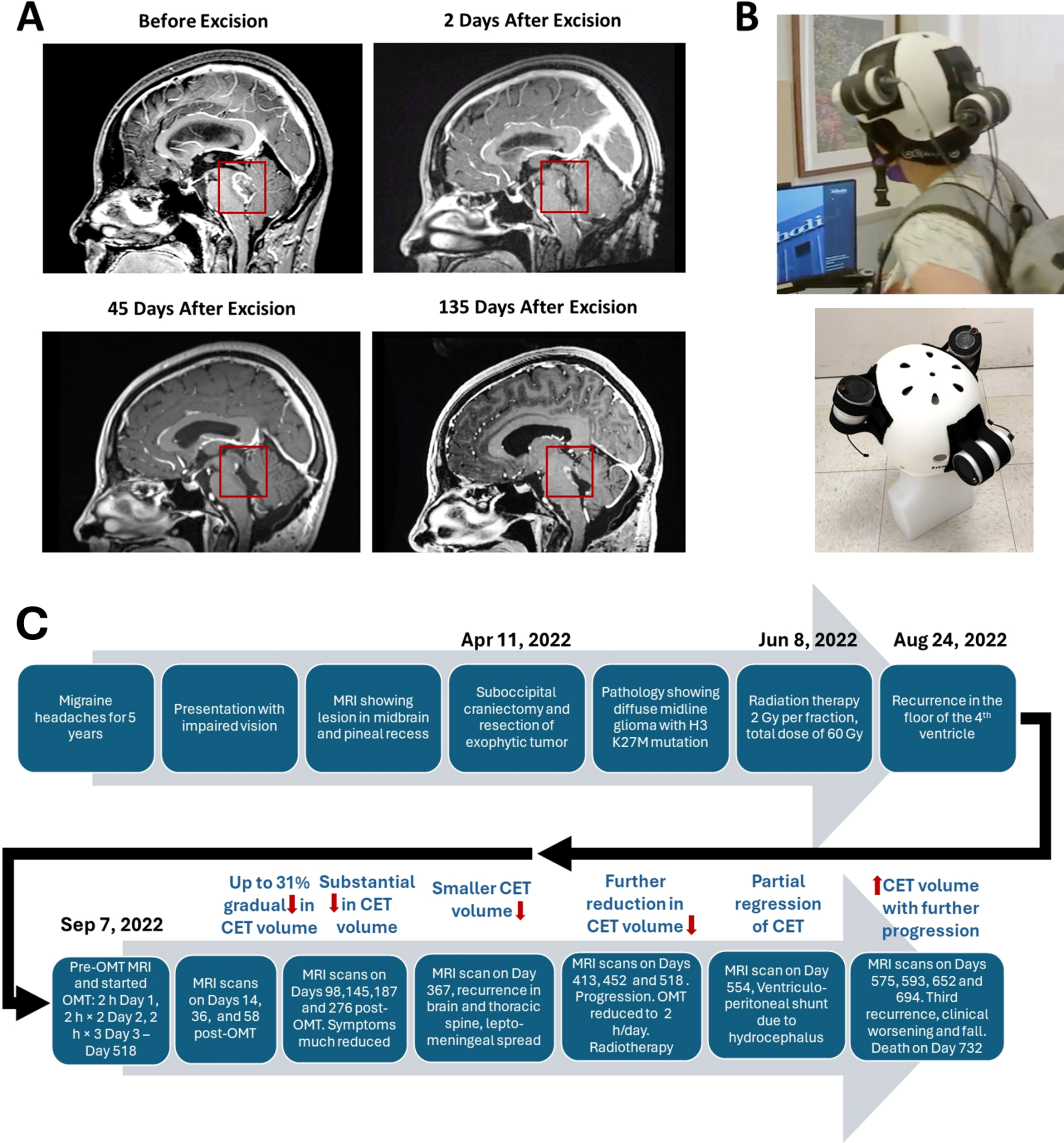
Contrast-enhanced tumor (CET) before and after surgical excision. **(A)**. T1-weighted post-contrast sagittal MRI images showing CET before, 2 days after and 45 days after tumor resection. An unresected residual CET is seen after surgery. **(B)**. Oncomagnetic Device Helmet. Top Subject wearing the Oncomagnetic Device helmet. Bottom Helmet placed on a dummy head illustrating the locations of the three oncoscillators. **(C)** Timeline of the clinical course and the course of treatment of the patient.

The patient is a 28-year-old woman who had a biopsy-proven midline glioma with the H3 K27M mutation, a uniformly lethal variant of a malignant glioma that is unresponsive to treatment. The patient has a history of migraine headaches for 5 years. She presented with a complaint that her vision was impaired and she perceived motion of stationary objects in her visual field. Magnetic resonance imaging (MRI) demonstrated a lesion in the midbrain and pineal recess, extending anteriorly into the thalamus and compressing the brainstem posteriorly (**Figure 1A**). She underwent a suboccipital craniectomy on April 11, 2022. The exophytic component of the tumor was resected, but the portion of the tumor invading into the brainstem was left in place to avoid new neurological deficits (**Figure 1A**). After surgery she experienced occasional vomiting and fever, which slowly resolved. She felt somewhat better but still had balance issues, relating to invasion of the tumor into the brainstem. She then underwent a course of radiation therapy to the affected area at 2 Gy per fraction for a total dose of 60 Gy. She also received temozolomide 75 mg/m^2^/day. Despite radiation treatment the tumor showed recurrence with extension into the floor of the fourth ventricle as seen in **Figure 1A** at 135 days post-surgery.

Because of lack of any standard of care options available to her, she was enrolled in an FDA-approved EAP treatment protocol using OMD. She signed an approved informed consent on August 8, 2022. The EAP treatment was carried out under a protocol approved by the Houston Methodist Research Institute Institutional Review Board.

### Oncomagnetic device

The OMD used in this study consisted of 3 sOMF generating oncoscillators securely attached to an acrylonitrile butadiene styrene helmet and connected to an electronic controller powered by a rechargeable battery (12). The positions and orientations of the oncoscillators were as shown in **Figure 1B**. The oncoscillator located at the back of the helmet was in the right position to expose the entire brainstem to an effective magnetic field strength determined by our *in vitro* studies on human DMG cells (17).

### Oncomagnetic treatment

As reported for treatment of GBM, the treatment involved intermittent sOMF stimulation using an optimally effective frequency profile and timing pattern (12). The first 3 days of treatment involved dose escalation conducted in our clinic under the supervision of the treating physician and the Principal Investigator (DSB) of this study. The treatment was for 2 hours on the first day with a 5-min break between the first and the second hour. On the second day, two 2-hour sessions were conducted with a 1-hour break between the sessions. The number of 2-hour sessions was increased to three on the third day. This regimen was then continued daily by the patient, unsupervised at home until Day 518, after which OMT was reduced to 2 hours once a day because of possible pseudoprogression and subsequently to 2 hours twice a day. The total duration of treatment was >2776 hours over >694 days. The patient was trained in the use and care of the device while she was treated in the clinic. She was instructed to maintain a daily log of the conduct of treatment, and any observations regarding adverse or treatment effects.

### Clinical and neuroimaging assessments

The patient underwent clinical evaluation by the treating physician on each of the initial three days of therapy at the clinic. Subsequent assessments occurred on Days 14, 36, 58, 98, 145, 187, 276, 367, 413, 452, 518, 554, 575, 593, 652, and 694 following the commencement of treatment. MRI scans were also conducted on these days. The Day 0 scan was performed before treatment on the first treatment day. A Siemens Magnetom Terra 7T scanner was used to conduct MRI scans until Day 518. After that they were performed on a Siemens Magneton Vida 3T machine because of the placement of a ventriculoperitoneal shunt. The scans involved T1 magnetization prepared rapid gradient echo (MPRAGE) protocol with and without gadolinium contrast, and T2-weighted-Fluid-Attenuated Inversion Recovery (T2-FLAIR), T2-weighted Turbo Spin Echo (T2-TSE), and proton magnetic resonance spectroscopy sequences (^1^H MRS, see **Supplementary Appendix**).

### Data analysis

Changes in CET volume and non-enhancing tumor infiltration and edema, respectively, were determined from post-contrast T1 anatomical and T2-FLAIR MRI scans at each time point during treatment. Post-contrast T1 scans were done at 5 min and 75 min after contrast injection to allow us to estimate washout and retention of contrast, corresponding to predominantly contrast-labeled active and necrotic tissues, respectively, by performing a modified form of treatment response assessment mapping (TRAM), developed previously by Mardor and coworkers (18). Evaluation of the treatment effect on 5-min CET was also done in accordance with the radiographic response assessment in neuro-oncology (RANO) criteria for clinical trials (19). The **Supplementary Appendix** includes further details about image processing, as well as data normalization, analysis, and presentation.

## Results

The patient was treated with OMD for more than 694 days. After the first three treatment days in the clinic, she self-administered daily 2-hour OMT 3 times a day. The treatment was reduced to once a day after Day 518 because of apparent pseudoprogression of the disease and thereafter it was increased to 2 hours twice a day. We present her clinical findings and MRI and ^1^H MRS data analyses below.

### Clinical observations

OMT was well tolerated by the patient. She reported no serious adverse effects. During treatment she reported transient mild itching and redness in the occipital region of the scalp around the healed incision, which lasted for 2–3 days and was diagnosed by the treating physician as contact dermatitis. He concluded that it may or may not be related to her wearing the device helmet, given that she was wearing a polyester skullcap underneath the helmet. The symptoms resolved themselves spontaneously. In terms of symptomatic improvements, at 98 days of treatment she stated that she had stopped having the occasional headaches and dizziness that she was suffering from before. On Day 367 clinical brain and spinal MRI scans done at follow up showed apparent recurrence of the tumor, leptomeningeal spread and a tumor nodule in the thoracic spine. On Day 518 further progression was observed because of which the patient received craniospinal radiation treatment (28.8 Gy, 16 fractions). The patient was brought to the emergency room unconscious on Day 554 when she was found to have developed hydrocephalus. A ventriculoperitoneal shunt was placed, and she was admitted to the neurological intensive care unit. After discharge she received rehabilitation treatment. She had an impaired short-term memory and unsteady gait. She discontinued treatment after apparent progression and injuries due to a fall thereafter, for which she was admitted to the hospital. She expired on Day 732. **Figure 1C** shows a timeline of the course of initial treatment and OMT, and evolution of the patient’s condition until her death.

### MRI results

**Figure 2:**
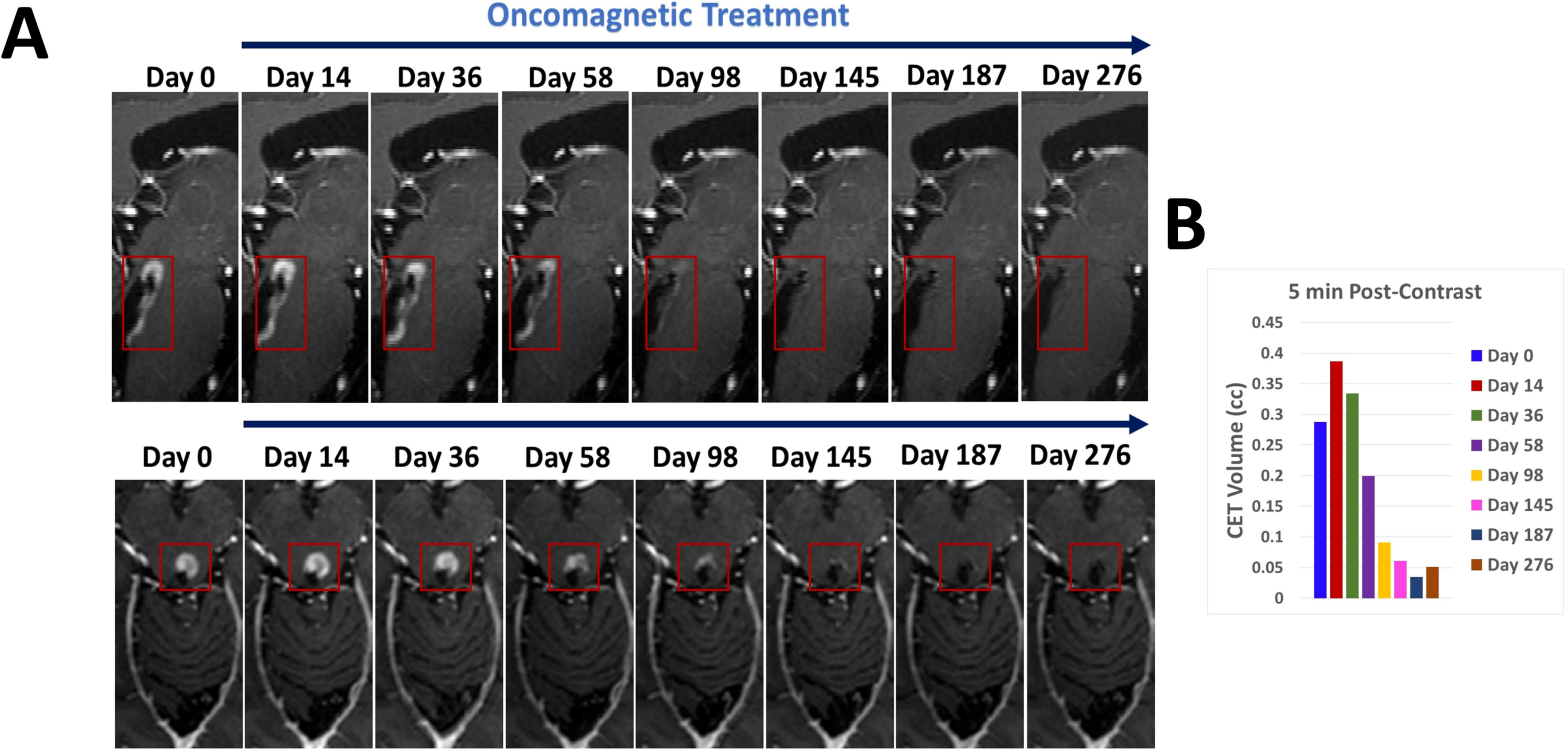
CET volume of recurrent tumor decreases during OMT. **(A)**. Segments of sagittal (top) and axial (bottom) T1-weighted post-contrast MRI scan slices illustrating the time course of reduction of CET volume over the course of OMT **(B)**. A bar plot showing the quantitative change in CET volume over time during treatment.

**Figure 3:**
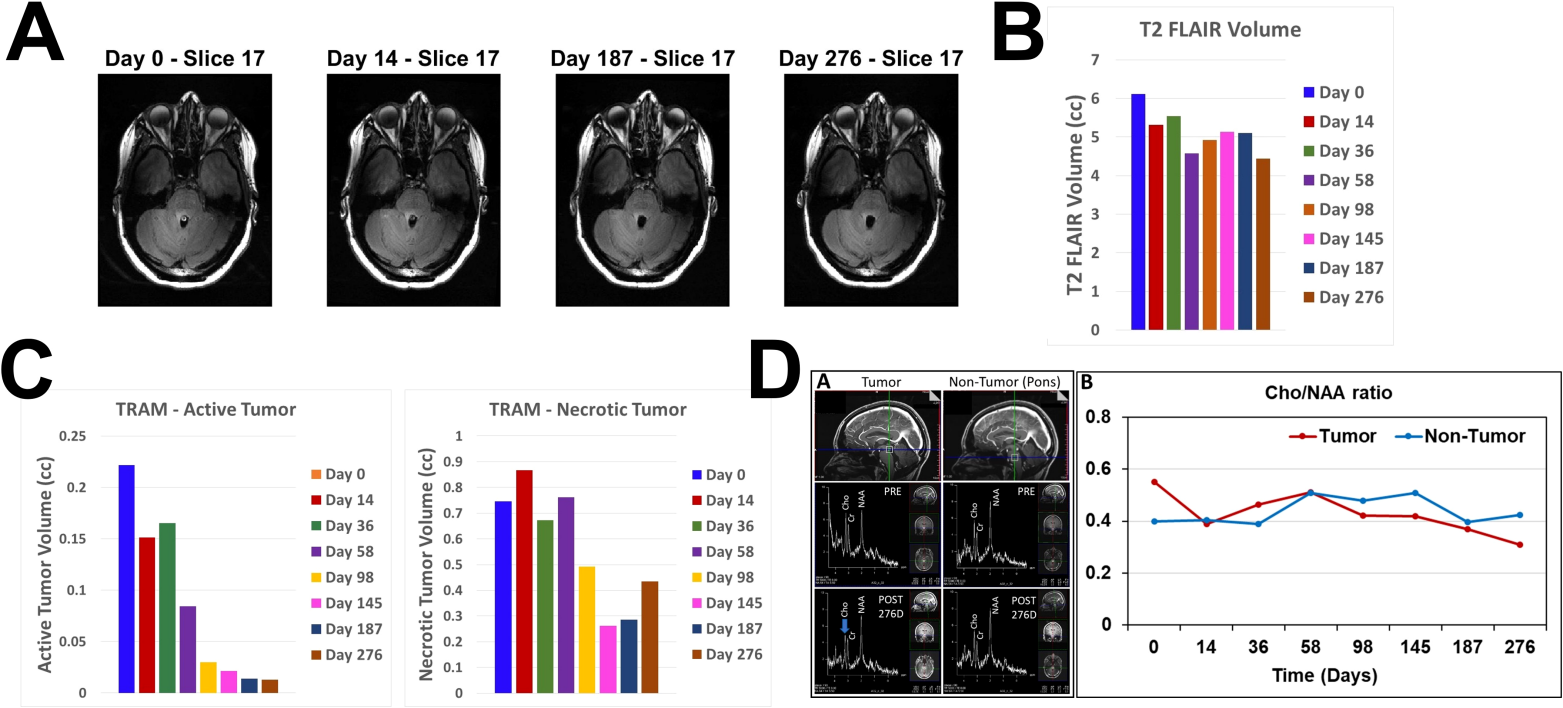
Changes in T2 FLAIR, estimated active and necrotic tumor tissues and ^1^H MRS during treatment. **(A)**. Representative axial slice images of MRI scans at 4 different time points obtained using T2 FLAIR pulse sequence **(B)**. Bar plot showing volumes of increased intensity in the T2 FLAIR scan images at all MRI scanning time points before and during OMT. **(C)**. Bar plots showing estimated volumes of active (left) and necrotic (right) tumor tissues at all MRI scanning time points before and during OMT. **(D)** sOMF treatment monitoring in the DIPG patient by *in vivo* ^1^H MRS on a 7T Siemens MAGNETOM Terra MRI scanner. Left – Sagittal views of the tumor and non-tumor regions showing the location of spectroscopic voxel (voxel size:12 x 12 x 12 mm3; upper panel), *in vivo* ^1^H MRS spectra obtained using the sLASER pulse sequence from the tumor and non-tumor regions before (PRE, middle panel), and after 276 days of sOMF therapy (POST 276D, bottom panel). Right – Plot showing choline-to-NAA ratio (Cho/NAA) in tumor and non-tumor regions at various time-points of sOMF therapy. It is worthwhile to note that choline signal intensity and Cho/NAA ratio were decreased in the tumor region during sOMF therapy.

Representative sagittal and axial images from 5-min T1 post-contrast scans and plotted data in **Figures 2A, B**, respectively, show gradual reduction in tumor volume with treatment over a period of 58 days. With 98 days of treatment there is a substantial reduction in CET. CET is almost completely absent on Day 145. The near-complete clearance of the contrast enhancement in the tumor is sustained and slightly further improved on Days 187 and 276. The slight increase in 5-min CET volume appears to be due to an increase in necrotic tumor tissue as explained below. Applying the radiographic RANO criteria we can conclude that the MRI scans showed stable disease until Day 58, a partial response on Day 98 and a near-complete response on Days 145, 187 and 276. The T2-FLAIR images showed a small volume of minimally elevated intensity pre-treatment and a 27% reduction of this volume on Day 276 of treatment (**Figures 3A, B**).

To estimate relative changes in contrast enhancement in active versus necrotic tumor tissues we subtracted the co-registered images at 75 min from those at 5 min post-contrast. Because of increased retention of the contrast in the extracellular space of necrotic tissues, negative difference values below a baseline threshold range are assumed to correspond to necrotic tissue and positive values above the baseline range to active tissue. The bar plots in **Figure 3C** show progressive decrease in active and necrotic tissue with treatment. There is a marked reduction of both estimated components of contrast enhancement with active tissue volume shrinking to <5% of the Day 0 volume on Days 145, 187 and 276. The small increase in necrotic tissue component on Day 276 suggests possible continued tumor tissue killing effect of OMT. Volume reductions of the 5-min CET and the estimated active and necrotic tissue components are seen in all axial slices as evident from **Supplementary Figures S1**–**S3** in the **Supplementary Appendix**.

**Figure 4:**
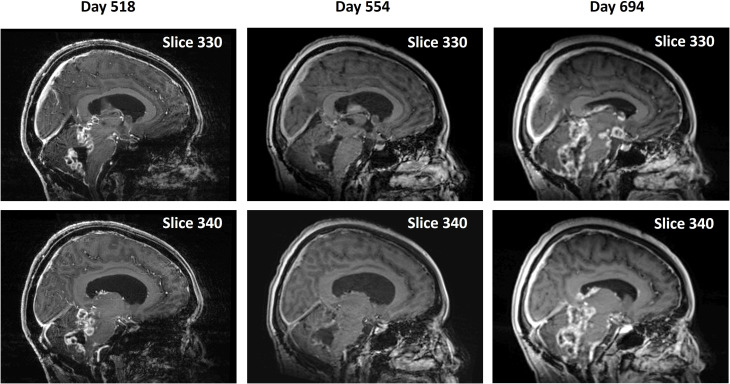
Second recurrence and remission of tumor during continued OMT and subsequent progression. Pairs of midline sagittal parasagittal T1-weighted post-contrast MRI scan slices during continued OMT showing second recurrence of the tumor on Day 518, its remission following OMT and radiation treatment on Day 554 and third recurrence on Day 694.

The near-complete absence of CET seen on Days 145 persisted until Day 367. However, from Day 367 through Day 518 there was progression of the disease with a wider recurrence of CET (**Figure 4**) for the second time, which showed partial regression on Day 554 with a combination of OMT and radiation treatment. A ventriculoperitoneal shunt was placed on Day 554 because of the presence of hydrocephalus. The patient continued receiving OMT subsequently beyond Day 694. An MRI scan done on Day 694 showed a third recurrence of the tumor (**Figure 4**). The treatment was discontinued ~ 3 weeks later after the patient had a fall and was admitted to the hospital for facial injuries. She expired on Day 732. **Supplementary Table S1** shows the timings and descriptions of events during the course of OMT. **Supplementary Table S2** shows changes in CET volume estimated from 5-min post-contrast MRI scans throughout the duration of treatment.

### ^1^H MRS findings

The effect of sOMF treatment on the tumor metabolism was also monitored by *in vivo* ^1^H MRS. **Figure 3D** left shows ^1^H MR spectra of tumor and non-tumor (pons) regions of the patient’s brain. Changes in the signal intensities of major metabolites N-acetyl aspartate (NAA), creatine (Cr) and choline (Cho) were monitored. ^1^H MRS showed a decrease in the signal intensity of Cho signal during OMT (**Figure 3D** left bottom panel, blue arrow). The Cho/NAA metabolic ratio is a measure of tumor proliferation index (MIB-1) (20). We determined this ratio in the tumor and non-tumor (pons) regions at different time intervals during treatment (**Figure 3D** right). The Cho/NAA ratio in the tumor region decreased by ~44% after 276 days of treatment. In the non-tumor region, this ratio showed no net decrease (**Figure 3D** right). Since Cho/NAA is a marker of tumor proliferation (MIB-1), a treatment-induced decrease in Cho/NAA suggests reduced tumor proliferation.

Although the near-complete absence of CET was seen after Day 145 continued until Day 367, there were multiple contrast-enhanced nodules detected in cerebellum, corpus callosum, and septal regions on Day 452 (**Supplementary Figure S4**). We monitored the effect of sOMF treatment on metabolic alterations in these newly formed lesions using ^1^H MRS. The Cho/NAA ratio was highest in the septal region (Cho/NAA = 1.89) compared to the other locations. This ratio was elevated (2.0 – 2.5) during the subsequent sOMF treatment (Day 518, 593, 652) and was decreased to 0.44 on Day 694. In addition, we also detected a gradual increase in the mobile lipid signals at 0.91 ppm (CH_3_ protons) and 1.32 ppm (CH_2_ protons) during sOMF treatment, which could be attributed to the treatment induced necrosis (from Day 518 to Day 652). On Day 694, the spectral profile of septal region resembled to that of the non-tumor region of the brain and the Cho/NAA ratio was in the range observed in the non-tumor region of the brain (0.49 ± 0.1; range 0.40 – 0.60) (**Supplementary Figure S4**). The presence of contrast-enhanced region and decreased Cho/NAA ratio may suggest pseudo-progression of the tumor.

## Discussion

Our results show that OMD-based sOMF therapy is well tolerated by a 28-year-old woman with untreatable recurrent DMG in the anterior wall of the 4^th^ ventricle extending into the midbrain. Remarkably, the treatment also causes an almost total regression of the CET after 145 days of treatment, consistent with a near-complete response in accordance with radiological RANO criteria. Previously, we have seen a similar reduction in CET volume in an end-stage recurrent GBM patient who was treated for 36 days with OMD (12). The current DMG patient continued receiving treatment and did not show any radiological signs of tumor recurrence until Day 367. Between Days 367 and 518 there was apparent tumor growth with leptomeningeal spread, which showed significant partial regression after OMT combined with radiation treatment. Arguably, the latter second recurrence might represent pseudoprogression (as indicated by evidence increased necrotic tumor tissue on MRS) that resolved with continued treatment. The patient continued receiving OMT for more than 694 days at which time an apparent third regression or pseudoprogression was observed. However, the treatment was discontinued shortly thereafter, and the patient could not return for a follow up MRI scan.

In contrast to our observations in the present case, the expected clinical course of DMG in adults is characterized by a median overall survival of 9–19 months, which is longer than in children (21). Recurrence or progression after radiation usually occurs near the original site and follows a pattern similar to pediatric tumors. Progression-free survival is also similar. Tumors originating in the brainstem generally show more rapid progression and infiltration. The longest documented survival for an adult with H3K27A brainstem DMG is 23 months (6). The patient in the present study survived for 30 months after diagnosis and tolerated OMT well. In addition, there was almost complete disappearance of the post-surgical contrast enhancing lesion with OMT. While symptoms such as fatigue, headaches, hydrocephalus, unsteady gait, memory loss, and falls were likely related to disease progression, OMT side effects may also have contributed.

Pre-clinical studies with cultured DMG cells in our laboratory have shown that sOMF causes substantial increase in intracellular ROS, leading to caspase-dependent apoptosis, in line with the effect on GBM cells (13, 22). As in the case of GBM *in vitro*, sOMF treatment also reduces their clonogenic survival by >60% (17). These findings indicate that the underlying mechanism of action of sOMF in DMG is analogous to that in GBM, involving disruption of electron transport in the mitochondrial respiratory chain, with release of ROS producing cancer cell oncolysis ().

There are no published studies on the use of any noninvasive device to treat this type of tumor. Electric field treatment in the intermediate frequency range, i.e., Optune^®^ has not been tested against this type of glioma. There are also no reports of any pre-clinical studies using electromagnetic stimulation as a treatment for DMG even in cell culture. Therefore, OMT is the first therapy of its kind that has shown a marked beneficial effect in this tumor *in vitro* and in this first patient case report. The near-complete reduction of CET with OMT at Day 145 and the subsequent partial remission after second recurrence or pseudoprogression suggests a possible effective immunological response and T cell-mediated clearance of necrotic tumor.

Current treatment options for DMG are limited and the prognosis with treatment is uncertain (23). Treatment involves surgery when possible and radiation therapy (23). No standard of care chemotherapy is recommended. It may be used by treating physicians on an individualized basis. To our knowledge, there is no report of radiation or chemotherapy causing any significant reduction in DMG tumor volume in the literature. Surgical excision is not a treatment option in most patients with DMG because of diffuse infiltration by tumor cells and the sensitivity of subcortical sites of occurrence of these tumors (24). The common pontine location is close to centers that control respiration, heart rate and blood pressure (23). Over 200 therapeutic trials with chemotherapeutic agents have not succeeded (25) because of problems stemming from resistance to treatment (3, 26) and drug penetration. This type of tumor is particularly resistant to chemotherapy (25). Combinations of radiotherapy with drug treatment have failed to prolong overall survival (25). This has been hypothesized to be due to the unresponsiveness of emerging cancer stem cells to treatment (3), in addition to drug and radiation treatment-resistant mutations (26).

Published evidence suggests that DMG may be immunologically cold (27). Studies have shown that there is minimal infiltration of activated T cells and myeloid cells, suggestive of diminished immune recognition (11, 28, 29). Immune checkpoint marker expression is also found to be low. Therefore, this tumor is less likely to respond to immune checkpoint inhibitors (27). OMT could possibly turn the tumor hot from an immunologic standpoint, allowing the T cells to attack and clear dead tumor cell debris created by the treatment. Our preliminary transcriptomic and imaging mass cytometry studies in a syngeneic mouse GBM model show an increased immune response to the tumor (Pandey et al., unpublished observations).

Some limitations of this study are, because it is a single patient case report, there is no control group with which to compare its findings, making it difficult to conclude that they are exclusively due to the treatment and not due to the natural course of the disease in the patient. It is also impossible to isolate the effect of OMT from the possible synergistic effect of combined craniospinal radiation therapy received during the period of partial remission after the second recurrence, although repeat radiation therapy is of limited value in this tumor, and there was almost complete disappearance of the T1 post-contrast lesion that recurred after surgery and initial radiation therapy. Another limitation is that 4 of the 5 co-authors of this paper have a conflict of interest because they are co-inventors of OMD, which has been licensed to a commercial entity.

## Conclusion

In this first-in-human case study, chronic noninvasive OMD-based sOMF stimulation appears to be highly safe and suggests the potential for an effective treatment for untreatable DMG. Furthermore, it is substantially different in terms of technology, mechanism of action and risk profile, from potential therapies that have failed in clinical trials, as well as those that are currently under investigation. Further studies involving large cohorts of patients are needed to support the results of this single patient case report and to shed better light on the safety and efficacy of OMT.

## Data Availability

The original contributions presented in the study are included in the article/**Supplementary Material**. Further inquiries can be directed to the corresponding authors.
