# Supplementary material for: Case Report: A new noninvasive device-based treatment of a mesencephalic H3 K27M glioma
Source: Front Oncol. 2025 Nov 5;15:1626516. doi: 10.3389/fonc.2025.1626516 (PMC12626803; doi:10.3389/fonc.2025.1626516)
Supplement: Supplementary file 1 [file DataSheet1.pdf]

Supplementary Appendix

Processing and Analysis of Magnetic Resonance Imaging (MRI) Data

Using the MRIcron (Chris Rorden, McCausland Center for Brain Imaging) software program DICOM images of the MRI scans were converted to the NifTi 3D volume format. At each time point during treatment post-contrast scans were co-registered with the pre-treatment Day 0 scan using Statistical Parametric Mapping version 12 (SPM 12) software (Institute of Neurology, University College London) running in the MATLAB (Mathworks, Natick, MA) environment on a Windows 11 laptop computer. Contrast-enhanced tumor (CET) volume was then estimated using an in-house MATLAB script and a uniform normalized intensity threshold. Line graphs of the mean volume and intensity were plotted as a function of the axial slice number at each time point. Bar graphs of the total volume in all slices were also plotted as a function of time. Similar plots were made of the estimated contrast enhancement attributed to active and necrotic tumor tissue by subtracting the 75-min post-contrast scan volume from the 5-min volume, as described under *Methods*. T2-FLAIR volumes during treatment were co-registered as above with the pre-treatment scan. The volume of enhanced T2-FLAIR intensity was then estimated by normalized intensity thresholding using the MATLAB script, yielding a bar plot of the enhanced intensity volume as a function of time.

In vivo ^{1}H magnetic resonance spectroscopy (MRS)

In vivo ^{1}H MRS data was collected from the patient during the course of sOMF treatment on a 7.0 T whole body clinical MRI scanner (MAGNETOM Terra, Siemens, Erlangen, Germany) with the 32-channel transmit/receive (Tx/Rx) head coil. T1-weighted anatomical images of the patient brain were acquired using a three-dimensional magnetization-prepared rapid gradient-echo (3D MPRAGE) with the following acquisition parameters: 256 slices, slice thickness = 1 mm, TR = 2500 ms, TE = 1.35 ms, inversion time = 1100 ms, field of view = 256 x 256 x 256 mm3 (scan time = ~5 min). These anatomical images were used to position the spectroscopy voxel. Single voxel ^{1}H MRS data were acquired from a 12 x 12 x 12 mm^3 voxel using the semi-adiabatic localization by adiabatic selective refocusing (sLASER) pulse sequence with VAPOR water suppression pulses[1,2]. B_0 shimming was performed using fast, automatic shim technique using echo-planar signal readout for mapping along projections, FAST(EST)MAP[2]. The following acquisition parameters were used: TR = 5000 ms, TE = 28 ms, number of averages = 64, bandwidth = 3000 Hz, data points = 2048. The time domain data were preprocessed using Tarquin[3] and peak fitting was performed using ACD labs software (Advanced Chemistry Development, Inc., Toronto, Canada) to determine peak areas of metabolites – N-acetyl aspartate (NAA), creatine (Cr) and choline (Cho)**.** Cho/NAA peak ratio was calculated in tumor and non-tumors regions.

References

1. Oz G, Deelchand DK, Wijnen JP, et al. Advanced single voxel (1) H magnetic resonance spectroscopy techniques in humans: Experts' consensus recommendations. NMR Biomed 2020:e4236. DOI: 10.1002/nbm.4236.
2. Deelchand DK, Henry PG, Joers JM, et al. Plug-and-play advanced magnetic resonance spectroscopy. Magn Reson Med 2022;87(6):2613-2620. DOI: 10.1002/mrm.29164.

3. Wilson M, Reynolds G, Kauppinen RA, Arvanitis TN, Peet AC. A constrained least-squares approach to the automated quantitation of in vivo (1)H magnetic resonance spectroscopy data. Magn Reson Med 2011;65(1):1-12. DOI: 10.1002/mrm.22579.

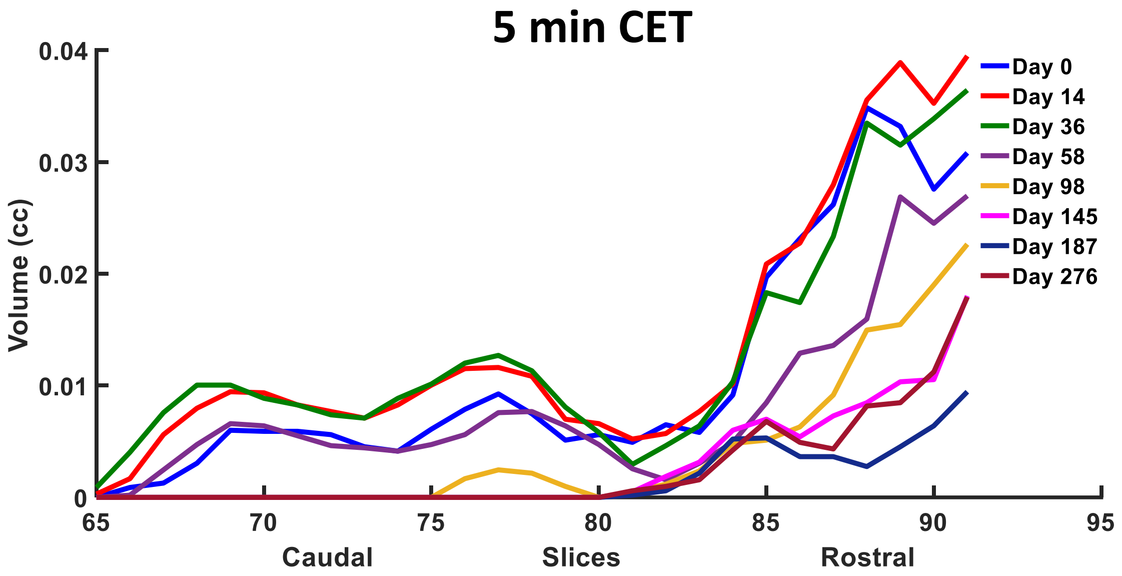

Figure S1

Contrast-Enhanced Tumor (CET) volume at 5 min post-contrast plotted as a function of axial slice number. The plotted lines represent scans conducted at different time points before and during treatment.

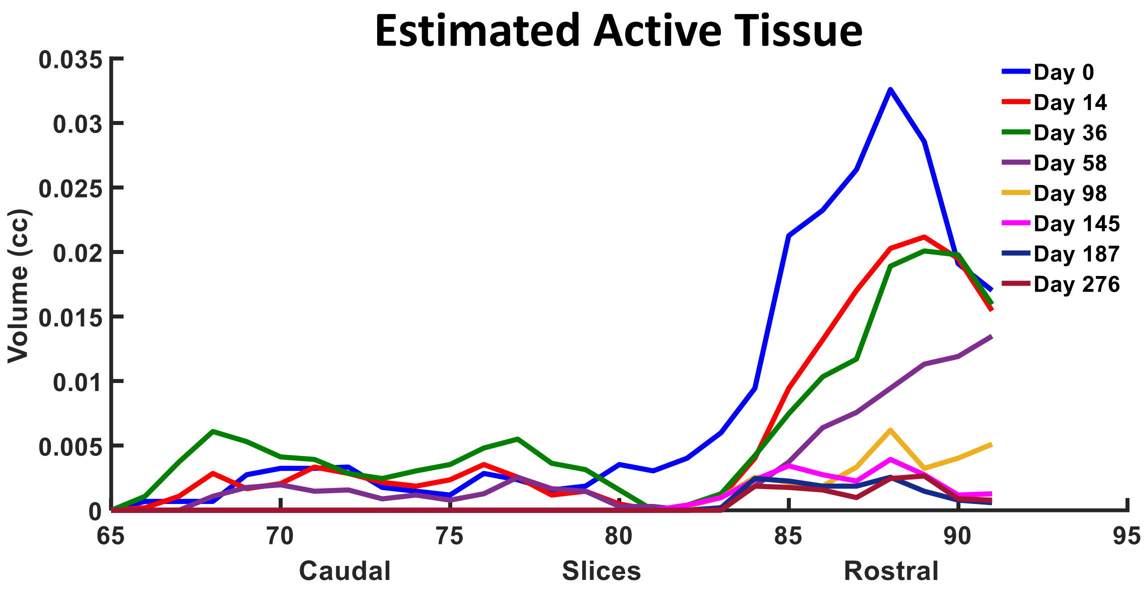

Figure S2

Estimated volume of contrast enhancement corresponding to active tumor tissue plotted as a function of axial slice number. The plotted lines represent scans conducted at different time points before and during treatment.

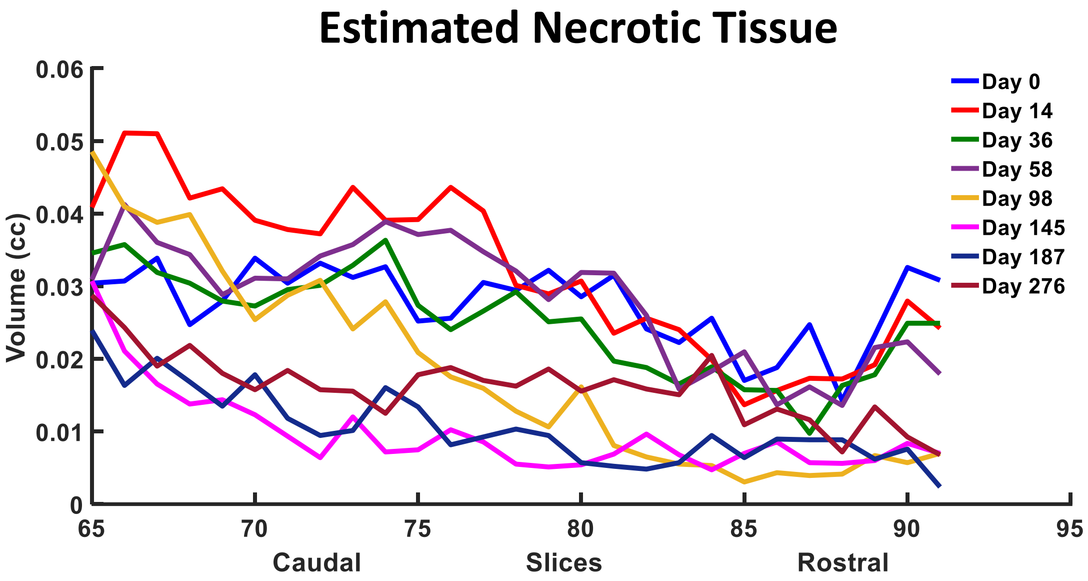

Figure S3

Estimated volume of contrast enhancement attributed to necrotic tumor tissue plotted as a function of axial slice number. The plotted lines represent scans conducted at different time points before and during treatment.

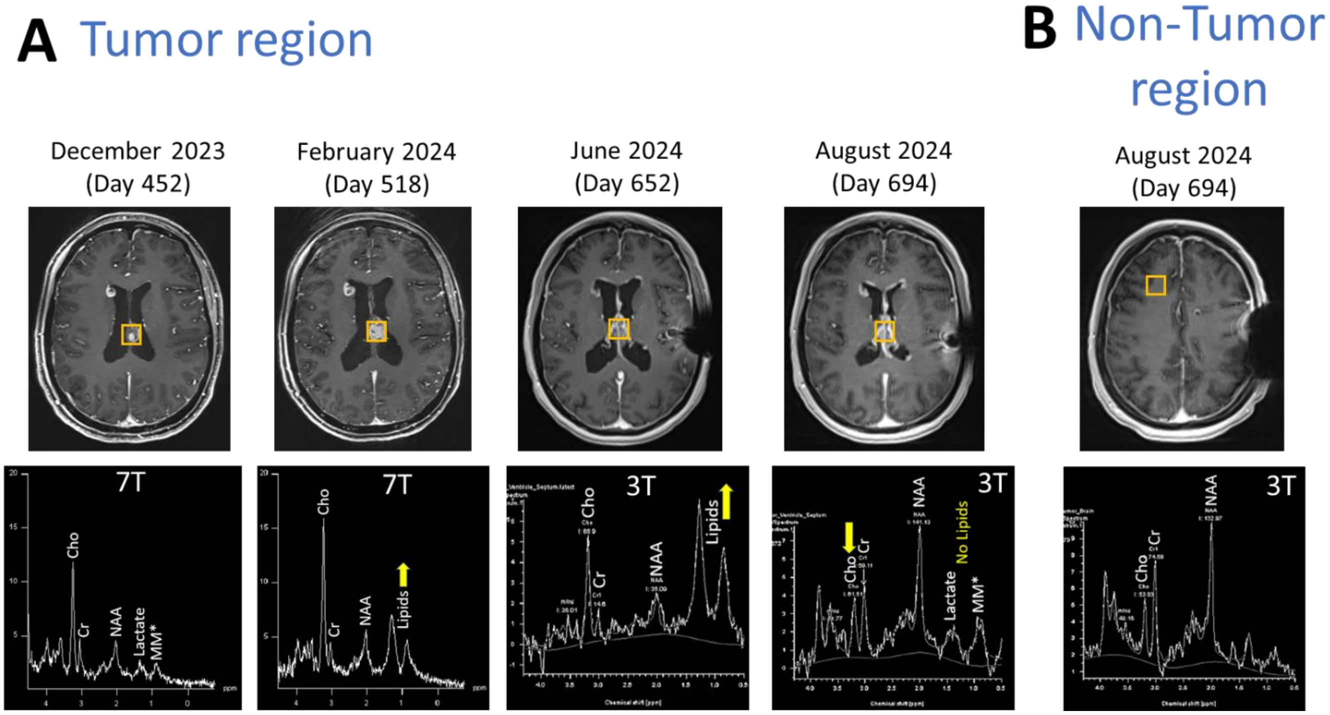

Figure S4

Treatment monitoring of newly formed recurrent tumor in the septal region using 1H MRS. (A) MR spectral profiles of newly formed lesion showing high choline (Cho) and low NAA peaks (Day 452). The presence of gradually increased mobile lipid signals (CH_3: 0.91 ppm and CH_2: 1.32 ppm) post-sOMF therapy suggests treatment-induced necrosis (Day 518 and 652). On Day 694, the spectral profile resembles that of normal brain tissue, shown in (B). Also, the disappearance of lipid signals on Day 694 suggests the clearance of necrotic debris. (*MM-amino acids signal from macromolecules).

Table S1

Date	Event	Comments	Dexamethasone
4/11/2021	Surgery Debulking		
	Pathology	DIPG H3 K27M	
6/8/2022	Started CRT	TMZ 75 mg/m^2/day	2 mg
7/2/2022	Visit	Dexamethasone stopped	0 mg
7/21/2022	Ended CRT		0 mg
8/24/2022	Progression		0 mg
9/7/2022	OMT started		0 mg
11/6/2022	Visit	Irritation of the back of the scalp from wearing the helmet, mild contact dermatitis	0 mg
10/10/2023	Visit	Recurrence with multiple nodules	
11/20/2023	Thoracic spine MRI	Tumor nodule in spine	
12/5/2023	Visit	Progressive disease with more nodules and leptomeningeal spread	
1/10/2024	Craniospinal radiation treatment started		2 mg twice a day
2/21/2024	Radiation treatment completed	Complains of fatigue	4 mg 4 times a day then taper.
3/18/2024	Left frontal VP shunt placement for hydrocephalus		1 mg thrice a day
4/26/2024	Visit	Improvement	2 mg twice a day
6/28/2024	Visit	Improvement continues	1 mg twice a day
8/13/2024	Visit	Progression	1 mg twice a day
9/20/2024	Patient expired	She was admitted for treatment of facial injuries due to a fall before her death	

Table S2 Changes in Contrast-Enhanced Tumor Volume in MRI Scans at 5-min Post-Contrast Time Point

Days Post-OMT	Day 0	Day 14	Day 36	Day 58	Day 98	Day 145	Day 187	Day 276	Day 367	Day 413	Day 462	Day 518	Day 554	Day 575	Day 593	Day 652	Day 694
Change in Tumor Volume (%)	0	34.1	15.9	-30.8	-68.4	-79	-88	-82	-76.4	-46.4	-22.4	129.1	-74.7	-63.2	170	145.1	203.7
OMT	2 h/day	2 h x 3/day	2 h x 3/day	2 h x 3/day	2 h x 3/day	2 h x 3/day	2 h x 3/day	2 h x 3/day	2 h x 3/day	2 h x 3/day	2 h x 3/day	2 h/day	2 h x 2/day	2 h x 2/day	2 h x 2/day	2 h x 2/day	2 h x 2/day
Other Intervention												Cranio-spinal radiation	Ventriculo-peritoneal shunt				
